# Basic studies on epigenetic carcinogenesis of low-dose exposure to 1-trichloromethyl-1,2,3,4-tetrahydro-β-carboline (TaClo) *in vitro*

**DOI:** 10.1371/journal.pone.0172243

**Published:** 2017-02-15

**Authors:** Renjie Wang, Yi Cui, Yi Xu, Joseph Irudayaraj

**Affiliations:** 1 College of Chemistry and Chemical Engineering, Chongqing University, Chongqing, China; 2 Department of Agricultural and Biological Engineering, Bindley Bioscience Center, Purdue University, West Lafayette, Indiana, United States of America; 3 International R & D Center of Micro-Nano Systems and New Materials Technolog, Chongqing University, Chongqing, China; 4 Key disciplines laboratory of Novel Micro-Nano Devices and System Technology, Chongqing University, Chongqing, China; 5 Microsystem Research Center, School of Optoelectronic Engineering, Chongqing University, Chongqing, China; Universidad de Chile, CHILE

## Abstract

1-Trichloromethyl-1,2,3,4-tetrahydro-β-carboline (TaClo) has been widely studied as a neurotoxic substance, however, only few reports have explored its effect on carcinogenicity. Since the aberrant modification of DNA methylation occurs very early in almost all human cancers, the focus of this study is to assess the carcinogenicity of TaClo by characterizing alterations of the epigenetic state, specifically, DNA methylation, upon exposure to TaClo in a HEK 293 model cell line. Our results suggest that TaClo could induce global DNA hypomethylation and transcriptional repression of critical tumor suppressor genes by increasing their promoter methylation. Enhanced cell proliferation, migration and anchorage independent growth were observed in cells exposed to TaClo. Our study highlights the epigenetic toxicity of TaClo, which contributes to its carcinogenicity by altering the DNA methylation status.

## Introduction

1-Trichloromethyl-1,2,3,4-tetrahydro-β-carboline (TaClo) is a well-known mammalian alkaloid [1], which originates in the human organism by Pictet—Spengler condensation of endogenously present biogenic amine tryptamine (Ta) and non-natural chloral hydrate (Clo) [2]. Humans are exposed to chloral hydrate from different sources. For example, chloral hydrate is widely used as a sedative and even as an anaesthesia-inducing agent [3], and it is also a useful laboratory chemical reagent and precursor. Moreover, chloral hydrate is a metabolite of some industrial solvents, such as trichloroethylene (TCE), which has been found in drinking water [4] or as an inhaling vapor pollutant [5]. Once inside the human body, chloral hydrate and amine tryptamine can spontaneously form TaClo [6]. Because its structure is closely related to 1-methyl-4-phenyl-1,2,3,6-tetrahydropyridine (MPTP) [7], a well-established synthetic neurotoxin, a number of studies involving TaClo have focused on its neurotoxic properties. For example, TaClo has been demonstrated to have negative effects on the growth of both glial [8] and serotonergic cells [9], and severely disrupts the extracellular serotonin and striatal dopamine metabolism [10, 11], which contributes to cell death of dopaminergic neurons and is involved in the development of idiopathic Parkinson’s disease [12].

In addition, Bringmann and colleagues [13] reported that TaClo inhibits catecholamine biosynthesis in a dose-dependent manner leading to a marked loss of tyrosine hydroxylase (TH) activity, which catalyzes the hydroxylation of l-4-hydroxyphenylalanine (l-tyrosine) to l-3,4-dihydroxyphenylalanine (l-DOPA) in the first step of catecholamine biosynthesis. The cytotoxicity of TaClo to neuronal-like clonal pheochromocytoma PC12 cells was assessed by Bringmann et al [14] by determining the activity of lactate dehydrogenase (LDH) relative to the total LDH activity in culture medium. The results showed that TaClo had a strong cytotoxic activity towards PC12 cells, and the ED50 value was 230 μM after 48 h incubation. This is probably because TaClo could severely inhibit the mitochondrial respiratory chain [15] and ultimately cause DNA damage and cell death [16].

Due to the high lipophilicity of the CCl_3_ group [17], TaClo is able to penetrate biological membranes passively [18]. This implies that the toxicity of TaClo is not specific to nerve cells or other related cells, but also plays a critical role in a myriad of health problems.

It has been proposed that long-term exposure to certain chemicals and physical hazards constitutes a main cause of chronic diseases, including cancer [19, 20], where aberrant DNA methylation is often considered as a hallmark [21]. For example, chronic TCE exposure has been suggested to be associated with hepatocellular carcinoma (HCC) and kidney cancer in humans [22]. Jiang et al discovered that the carcinogenic mode of action of TCE is due to the aberrant transcriptional changes caused by both hypo- and hyper-methylation on the promoter regions of single-copy genes [23]. In our recent work we showed that TCE could disrupt the *Dnmt3a* -DNA association to induce global DNA hypomethylation [5].

In general, cancer cells are characterized by global hypomethylation and gene-specific hypermethylation. A larger number of studies demonstrated exposure to carcinogenic metals, such as nickel [24], arsenic [25], cadmium [26], and chromium (VI) [27], could induce global hypomethylation as well as promoter hypermethylation of specific tumor suppressor genes, inducing the methylome like cancer cells [28]. So far, the cytotoxicity and neurotoxic properties of TaClo were studied by a large number of researchers, however there are no published reports on the carcinogenicity of TaClo.

In this study, we investigated the carcinogenic potential of TaClo by evaluating DNA methylation modifications after exposure to TaClo. Since global DNA hypomethylation occurs quite early in human cancers [29], and promoter hypermethylation of tumour suppressor genes [30] can play an important role in carcinogenesis [31], we first performed genome-wide global methylation analysis in HEK 293 cells treated with TaClo at extremely low concentration (10 ppb) for a four week exposure (the half-maximal toxic concentration value of TaClo to HEK 293 cells was 7 ppm [32]). The promoter methylation status of 22 tumor suppressor genes as well as 11 CpG sites in *BRCA1* promoter region was then examined. Finally, changes in expression levels of the hypermethylated genes were investigated. Our results showed that global DNA methylation significantly decreased and the transcriptional levels of DNA demethylase (i.e., ten-eleven translocation TET proteins) increased in TaClo-treated cells. Furthermore, the promoter regions of critical tumour suppressor genes were hypermethylated, and as expected, the expressions of these genes were repressed in cells. We further observed that the treated cells were overactive in proliferation and migration rate because of the aberrant DNA methylation and the anchorage independent growth ability was enhanced after exposure to TaClo. Altogether, our study provides basic overview of DNA methylation and carcinogenicity due to TaClo exposure *in vitro*, providing a strong basis for *in vivo* exploration.

## Materials and methods

### Chemicals

TaClo (>99.5% pure, CAS 6649-90-7) was purchased from Exclusive Chemistry Ltd (Kaluga region, Obninsk, Russia). Paraformaldehyde (PFA, CAS: 30525-89-4), Triton X-100 (CAS 9002-93-1), hydrochloric acid (HCl, CAS: 7647-01-0), bovine serum albmin (BSA, CAS: 9048-46-8) and phosphate buffered saline (PBS) were purchased from Thermo Fisher Scientific Inc. Waltham, MA, USA. Normal goat serum was purchased from Jackson ImmunoResearch Inc. West Grove, PA, USA.

### Cell culture and treatment with TaClo

HEK 293 cells were cultured in dulbecco’s modified eagles medium (DMEM; Thermo Fisher Scientific Inc. Waltham, MA, USA) supplemented with 10% heat-inactivated fetal bovine serum (FBS), 1% penicillin-streptomycin (Thermo Fisher Scientific Inc. Waltham, MA, USA), and incubated in 37°C with humidified air containing 5% CO_2_. For TaClo treatment, TaClo was added into the medium and the final concentration was adjusted to be 10 ppb. TaClo exposure lasted for 4 weeks, and a parallel cell culture without TaClo was used as control group. Cells were harvested and regrowth every 2–3 days (at 70–80% confluency) and sub-cultured.

### Determination of genomic global DNA methylation

Genomic DNA of TaClo treated and control cells were extracted and purified with DNeasy kit (Qiagen Inc., Germantown, MD, USA) according to manufacturer’s instructions. And an additional RNase A treatment was adopted as recommended by the instructions. The concentrations of extracted DNA were measured by a NanoDrop spectrophotometer (Thermo Fisher Scientific Inc., Waltham, MA, USA). Genomic DNA methylation (5mC) level in both the control and TaClo treated cells were determined with MethylFash Methylated DNA Quantification Kit (Epigentek Inc. Farmingdale, NY, USA) following manufacturer’s protocol. In the assay, 100 ng of DNA was bound to each strip well specifically treated to have high DNA affinity. The methylated fraction of DNA was detected through an ELISA-like reaction by using capture and detection antibodies and then quantified by reading the absorbance (at 450 nm) using SpectraMax plus microplate reader (Molecular Devices, LLC., Sunnyvale, CA, USA). The amount of methylated DNA was proportional to the OD intensity measured, which can be calculated with the formulas included in the kit to assess the relative methylation status of different DNA samples.

### Immunostaining and fluorescence images

5-azacytidine (Sigma-Aldrich Co. LLC. St.Louis, MO, USA) was used as positive control in this immunofluorescence experiment. HEK 293 cells were cultured onto glass coverslips and treated with 5μM 5-azacytidine (5-AZA) for 8 days. The media containing 5-AZA was changed daily. Then the control HEK 293 cells, TaClo and 5-AZA treated cells were fixed with 4% paraformaldehyde (PFA) solution for 15 min at 4°C. Cells were then washed with PBS and permeabilized in 0.4% Triton X-100 PBS solution for 30 min with gentle shaking. After PBS wash, the permeabilized cells were denatured with 2N HCl at 37°C for 20 min and neutralized in Tris-HCl-100 for 10 min at 4°C. Then the cells were blocked with normal goat serum solution containing 1% BSA for another 60 min, and incubated with DNA methylation mark targeting primary antibodies (mouse monoclonal, lot: 101110; Eurogenetec, CA) overnight on a shaker. After washing with PBS, the cells were incubated with goat anti-mouse secondary antibodies for 60 min, and then washed with PBS three times. Images were acquired using the Zeiss immunofluorescence microscope Axiovert 200 and Axiovision software.

### Tumor suppressor genes DNA methylation PCR array and pyrosequencing assay

The promoter methylation pattern of tumor suppresser genes was analyzed using Methyl-Profiler DNA Methylation PCR Array Systems (SAbioscience-Methyl-Profiler^™^ DNA Methylation PCR Array System, 96-well set-up, Qiagen Inc., Germantown, MD, USA), which could detect the DNA methylation status at CpG islands of cellular genes accurately. This promoter methylation assay system relies on two different restriction endonucleases (Methylation-sensitive enzyme A and Methylation-dependent enzyme B) which can cleave the target sequences either in the presence or absence of methylated cytosine. The assay was performed according to manufacturer’s instructions. In brief, DNA collected from control and TaClo treated HEK 293 cells were digested with the mentioned two different restriction endonucleases. The relative amount of DNA remaining after each enzyme digestion was quantified with real-time PCR. Data analysis was based on the algorithm provided by the manufacturer.

For pyrosequencing assay, 500 ng DNA samples were bisulfite treated using a proper bisulfite salt solution by EpigenDx company (D5004). Then the treated DNA were diluted to 45 μL and 3N NaOH (5 μL) was added. DNA denaturation was carried out by incubating the samples at 42°C for 30 minute. 100 μL of bisulfite salt solution was added to the DNA and incubated at 50°C for 14 hours. Zymogen DNA columns and 20 μl of T_1_E_0.2_ pH 8.0 was used to purify and elute bisulfite treated DNA. PCR was performed using 1 μl of the final DNA sample and 0.2 μM of each primer. Biotinylated PCR primers and Sepharose beads were used to purify the final PCR product. The PCR product was bound to Streptavidin Sepharose HP (GE Healthcare Life Sciences, Pittsburgh, PA, USA), and the Sepharose beads containing the immobilized PCR product were purified, washed and denatured using a 0.2 M NaOH solution and rewashed using the Pyrosequencing Vacuum Prep Tool (Pyrosequencing, Qiagen Inc., Germantown, MD, USA) as recommended by the manufacturer. Then 0.2 μM pyrosequencing primers were annealed to the purified single-stranded PCR product. The PCR products (10 μL) were sequenced by Pyrosequencing PSQ96 HS System (Pyrosequencing, Qiagen Inc., Germantown, MD, USA) according the manufacturer’s instructions. The methylation status of each *locus* was analyzed individually as a T/C SNP using QCpG software (Pyrosequencing, Qiagen Inc., Germantown, MD, USA).

### Quantitative real-time PCR (qRT-PCR)

Total RNA was extracted from the control and TaClo treated cells using the RNeasy mini kit (Qiagen Inc., Germantown, MD, USA) and reverse transcripted to cDNA using the iScript cDNA synthesis kit (Bio-Rad Laboratories, Inc., Hercules, CA, USA). The changes of target genes expression were determined using 20 uL of SYBR Green PCR master mix (Thermo Fisher Scientific Inc., Waltham, MA, USA) in a quantitative real-time PCR system (StepOnePlus Real-Time PCR Systems; v 2.0 Applied Biosystems, USA). Amplification conditions were 95°C for 1 min, followed by 40 cycles at 95°C for 15 second and 60°C for 1 min. Data analysis was based on ΔΔC_t_ method with normalization of transcription to the *GAPDH* gene. Primer sequence of the target genes have been summarized in Table 1.

**Table 1:** qRT-PCR primers used in this study. All primers listed are from 5’ to 3’.

*GAPDH*	(F) CAGCCTCAAGATCATCAGCA
	(R) TGTGGTCATGAGTCCTTCCA
*Dnmt3a*	(F) TATTGATGAGCGCACAAGAGAGC
	(R) GGGTGTTCCAGGGTAACATTGAG
*Dnmt3b*	(F) GGCAAGTTCTCCGAGGTCTCTG
	(R) TGGTACATGGCTTTTGGATAGGA
*Dnmt1*	(F) TACCTGACGACCCTGACCTC
	(R) CGTTGGCATCAAAGATGGACA
*TET1*	(F) AATGGAAGCACTGTGGTTTG
	(R) ACATGGAGCTGCTCATCTTG
*TET2*	(F) AATGGCAGCACATTGGTATG
	(R) AGCTTCCACACTCCCAAACT
*TET3*	(F) ATGTACTTCAACGGCTGCAA
	(R) CGGAGCACTTCTTCCTCTTT
*P53*	(F) CCCCTCCTGGCCCCTGTCATCTTC
	(R) GCAGCGCCTCACAACCTCCGTCAT
*CDKN2A*	(F) GAGGCCGGGATGAGTTGGGAGGAG
	(R) CAGCCGGCGTTTGGAGTGGTAGAA
*BRCA1*	(F) CAAGGAACCAGGGATGAAATCAG
	(R) ATGGCTCCACATGCAAGTTTG
*DAPK1*	(F) GGGTGTTTCGTCGATTATCAAGA
	(R) TCGCCCATACTTGTTGGAGAT
*PTEN*	(F) TTTGAAGACCATAACCCACCAC
	(R) ATTACACCAGTTCGTCCCTTTC
*RARB*	(F) CGTGGAGTTTGCTAAACGTCT
	(R) TGGTGTCTTGTTCTGGGGTAT
*FHIT*	(F) ATCTCATCAAGCCCTCTGTAGT
	(R) GGACGCAGGTCATGGAAGC
*CDH1*	(F) TACGCCTGGGACTCCACCTA
	(R) CCAGAAACGGAGGCCTGT
*CDH13*	(F) AATCCACAAACAAGCTGTTCC
	(R) GTTTCCCTGAATCTGTCACCA
*GSTP1*	(F) TTGGGCTCTATGGGAAGGAC
	(R) GGGAGATGTATTTGCAGCGGA
*PDLIM4*	(F) CGGAACCTCAAGCCCACG
	(R) CATGAAGCACTCGGGATGGT

### Cell proliferation and migration assays

Cell proliferation assays were performed in 6-well plates as previously reported with minor modifications[33]. Equal numbers (40000 cells) of HEK 293 cells with and without TaClo treatment were seeded into 6-well plate with three replicates respectively. Cell number of every well was detected every day.

For migration assay, cells were seeded in a 6-well culture plate and grown to confluence. Then a straight line ‘‘scratch” was induced in the cell monolayer with a sterile p200 pipette tip. Cell debris in the edge of the scratch was removed by washing with culture medium. The plate was then placed in a cell culture incubator for 18 h, and images at different time periods were taken using the EVOS FL cell imaging system (Thermo Fisher Scientific Inc., Waltham, MA, USA).

### Anchorage independent growth assays

CytoSelect^™^ 96-well cell transformation assay kit (Cell biolabs, Inc., San Diego, CA, USA) was used for the anchorage independent growth assays. Experiments were performed according to the specifications in the product manual. Briefly, equal volumes of 1.2% agar solution and 2X DMEM/20% FBS medium were mixed and 50 μL of the mixture was transferred to each well of a 96-well microplate immediately to evenly cover the wells. To solidify the base agar layer, the plate was transfered to 4°C for 30 min. HEK 293 cells were collected and suspended with and without TaClo treatment in culture medium at 2 x 10^5^ cells mL^-1^, and mixed with equal volumes of 1.2% agar solution and 2X DMEM/20% FBS medium (1:1:1). Then 75 μL of the mixture was transferred to the corresponding well of the 96-well microplate containing the solidified base agar layer (five replicates were used) and 100 μL of culture medium was added to each well and the cells were incubated for 8 days at 37°C at 5% CO_2_. At the end of this incubation, 50 μL of agar solubilization solution was added to each well to dissolve the agar completely followed by addition of 25 μL of 8X lysis buffer to each well. Then 10 μL of the mixture was used to react with 90 μL of the CyQuant working solution. The fluorescence intensities of different samples were tested by using Synergy NEO B multi-mode microplate reader (BioTek Instruments, Inc., Winooski, VT, USA) with a 485/520 nm filter.

### Statistical analysis

Student t-test was performed in this study for statistical analysis, and a threshold of p < 0.05 was considered statistically significant.

## Results

### TaClo exposure induces global DNA hypomethylation

**Fig 1:**
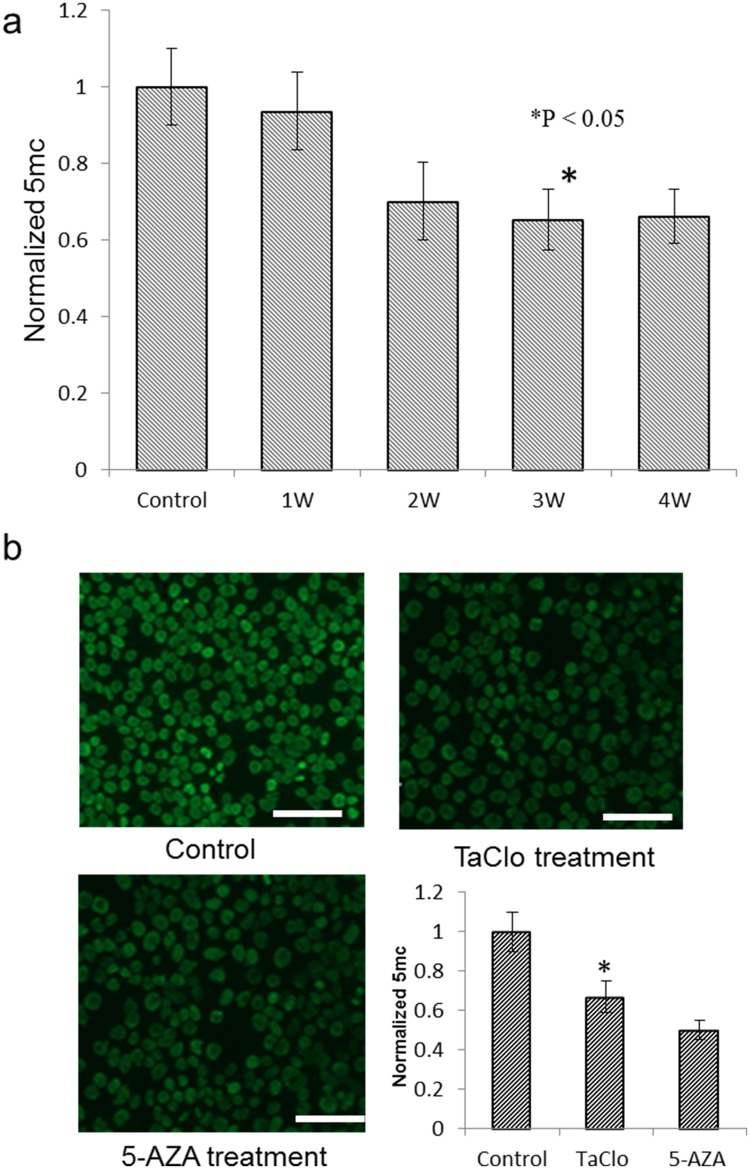
Low dose exposure to TaClo decreased global DNA methylation. (a) Global DNA methylation level detected at different time period indicates a gradual decrease due to TaClo exposure. (b) Immunofluorescence images of global DNA methylation of HEK 293 cells treated under different conditions. n = 4 independent replicates. Scale bar, 400 μm.

The global DNA hypomethylation is one of the most frequent and earliest epigenetic alterations observed in oncogenesis. To determine whether TaClo treatment could induce the loss of global DNA methylation, we first investigated the global DNA methylation level of TaClo treated cells at different time points. The results shown in Fig 1a indicated that the global DNA methylation level gradually decreased after one week of TaClo treatment, and a significant decrease was observed after three weeks. Compared to the control cell, the percentage of global DNA methylation level in cells treated with low concentration (10 ppb) of TaClo reduced by more than 30%. Meanwhile, there was no significant change in the level of global DNA methylation among the cells after three or four weeks’ treatment. Then HEK 293 cells treated with TaClo for 4 weeks were chosen for further experiments. Result of immunofluorescence for global DNA methylation confirmed the global demethylation effect (Fig 1b). The 5-AZA as a hypomethylating agent is approved by the U.S. Food and Drug Administration [34] and was used as positive control in this study. In comparison with the fluorescence images of control samples, the fluorescence intensities in both TaClo and 5-AZA treated samples significantly decreased around 30% and 40% respectively.

### Impact of TaClo treatment on *Dnmts* and *TETs* expression

**Fig 2:**
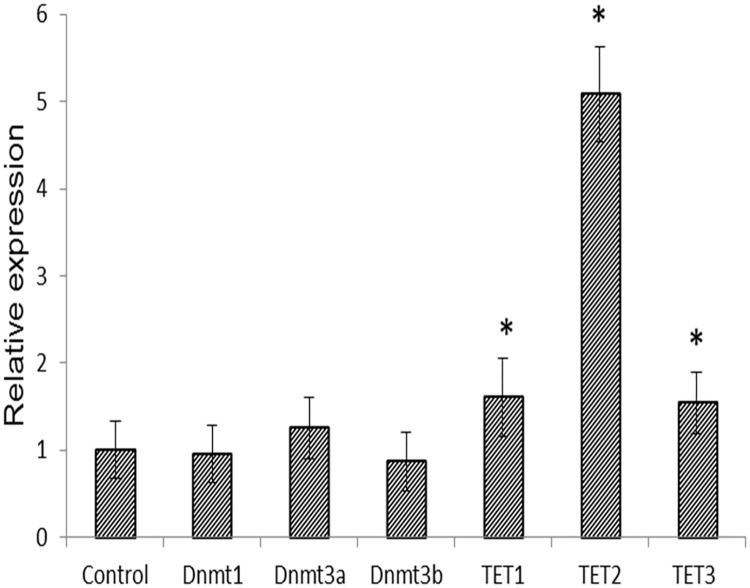
qRT-PCR analysis of *Dnmts* and *TET*s expression changes after TaClo treatment. The expression of *Dnmts* had no significant change, meanwhile the expression of *TETs* experienced a significant increase after TaClo treatment. (n = 4 independent replicates).

The function of DNA methyltransferases (*Dnmts*), including *Dnmt1*, *Dnmt3a*, and *Dnmt3b*, are to add a methyl group onto the carbon-5’ position of cytosine [35]. In contrast, *TET* proteins play an opposite role to remove the methyl marks [24]. Therefore the DNA methylation state has a very intimate relationship with the *Dnmts* and *TETs* expression. In this study, qRT-PCR was used to detect the transcriptional changes of *Dnmts* and *TETs* after TaClo treatment. As shown in Fig 2, the expression of all of the *TETs* experienced a significant increase, over 1.5-fold for *TET1* and *TET3* and over 5-fold for *TET2*. However, no significant change (< 1.5-fold) was observed for the *Dnmts*.

### TaClo exposure causes promoter hypermethylation of some tumor suppressor genes

**Fig 3:**
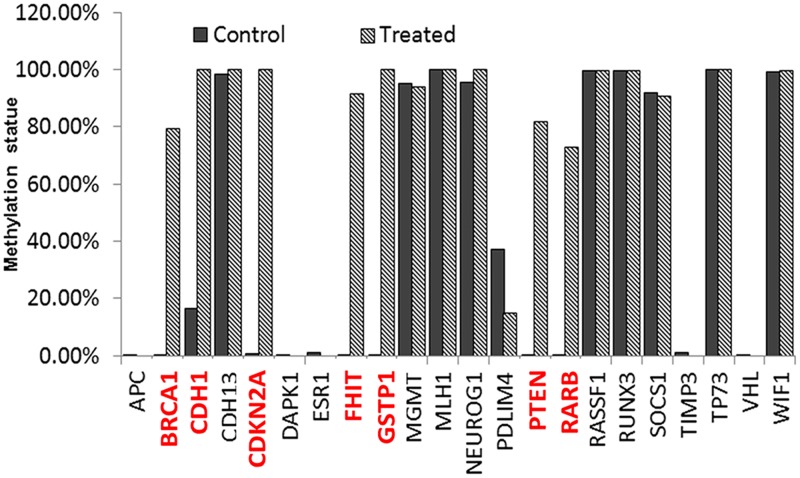
Promoter hypermethylation of selected tumor suppresser genes was observed after low dose exposure to TaClo.

Promoter hypermethylation of tumor suppressor gene is the most common aberrant epigenetic pattern and has been observed in various tumors [36]. Carcinogenic substances have been shown to cause genome-wide hypomethylation, while at the same time, can turn off some tumor suppressor genes by inducing promoter hypermethylation [37]. The objective of this study was to determine whether TaClo has an oncogenic effect under low concentration (10 ppb) exposure for a prolonged period to time. Since global hypomethylation has been demonstrated in this study, next we hypothesized that TaClo exposure could lead to promoter hypermethylation of tumor suppressor genes with DNA methylation PCR array. The methylation levels of 22 gene-promoters whose hypermethylation have been reported in the literature to occur frequently in a variety of cancers were evaluated. As expected, the results demonstrated that one third of these detected tumor suppressor genes (red-marked genes) in TaClo treated samples were highly methylated compared to the control group sample (Fig 3).

**Fig 4:**
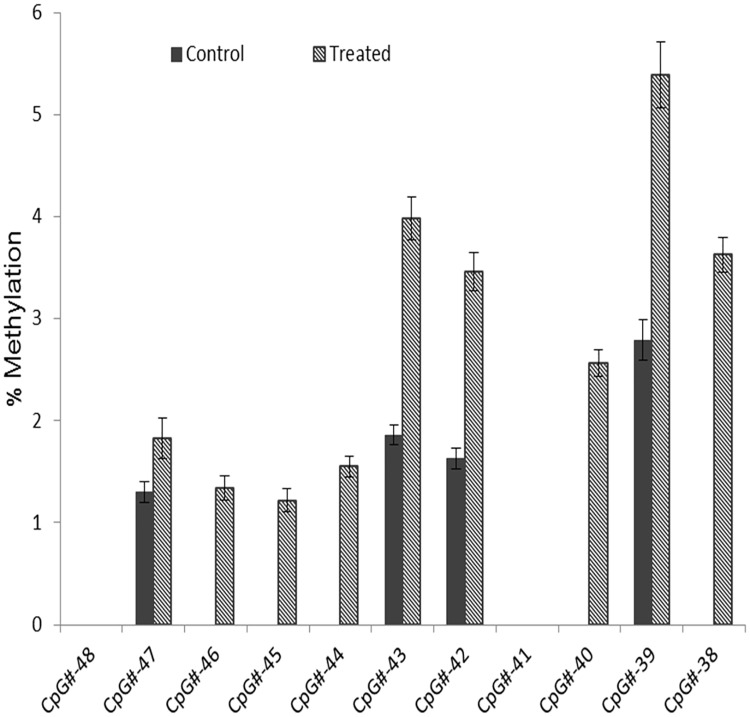
Increased methylation level of selected CpG sites in *BRCA1* promoter region induced by lower concentration of TaClo exposure for a prolonged period of time.

To further validate the promoter hypermethylation pattern of a representative tumor suppressor gene, methylation changes of 11 CpG sites from CpG#-48 to CpG#-38 in the *BRCA1* promoter region were quantitatively determined by pyrosequencing assays. Methylated CpG sites at selected *BRCA1* promoter region were either absent or present at very low levels in normal control HEK 293 cells, whereas methylation levels in 9 of 11 detected CpG sites increased in TaClo treated cells, of which 5 CpG sites (#-46, #-45, #-44, #-40, #-38) were *de novo* methylated after TaClo treatment. No methylation was detected in #-48 or #-41 CpG sites before or after treatment. Changes in the methylation status of individual CpG sites in the *BRCA1* promoter are shown in Fig 4.

### TaClo exposure leads to transcriptional repression of tumor suppressor genes

**Fig 5:**
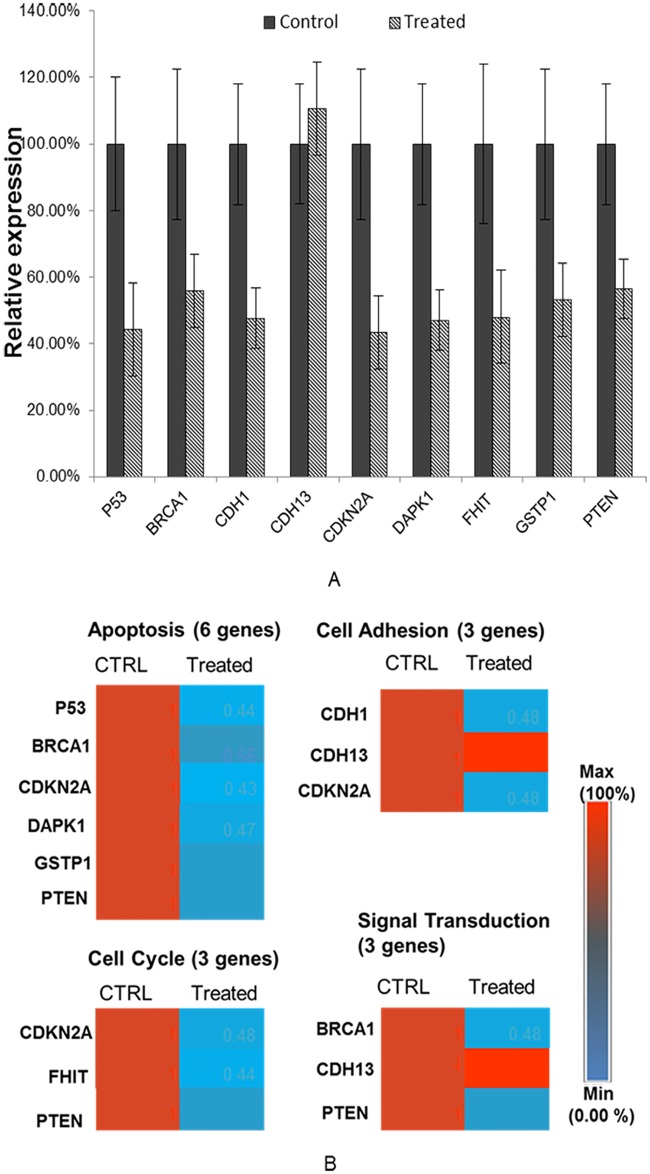
qRT-PCR analyses results of selected tumor suppressor gene expression. (a) Expression of promoter hypermethylated tumor suppressor genes significantly decreased. (b) Heat map for the transcriptional changes of promoter hypermethylated tumor suppresser genes.

It has been established unequivocally that promoter hypermethylation cause transcriptional silencing of a wide range of genes [38]. In order to corroborate the promoter hypermethylation profile induced by TaClo treatment into RNA transcriptional profile, 7 genes whose promoters were hypermethylated after TaClo exposure and 2 well-known tumor suppressor genes (*CDH13* and *P53*) were subjected to qRT-PCR analyses subsequently. The analyzed genes are involved in different functions, such as apoptosis (*P53*, *BRCA1*, *CDKN2A*, *DAPK1*, *GSTP1*, *PTEN*), cell adhesion (*CDH1*, *CDH13*, *CDKN2A*), cell cycle (*CDKN2A*, *FHIT*, *PTEN*) and signal transduction (*BRCA1*, *CDH13*, *PTEN*). The qRT-PCR results were analyzed and presented in Fig 5. The results demonstrated that the expression of these tumor suppressor genes significantly decreased compared to the control group, and the transcriptional profiles of the *CDH13* gene, whose promoter was not hypermethylated after TaClo treatment, had no significant changes. These findings indicated that TaClo exposure can cause transcriptional repression of tumor suppressor genes by elevating the promoter methylation.

### TaClo exposure promoted cell proliferation and migration

**Fig 6:**
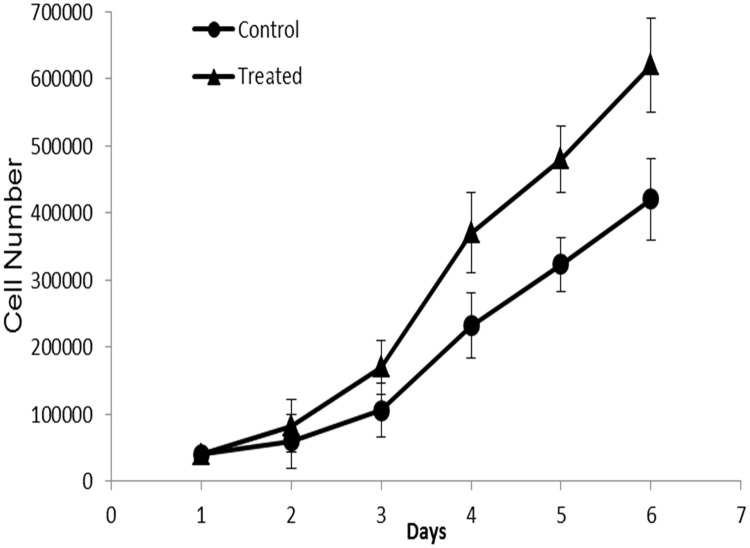
Growth curves of control and TaClo treated HEK 293 cells. Statistically significant difference was observed in day 6.

**Fig 7:**
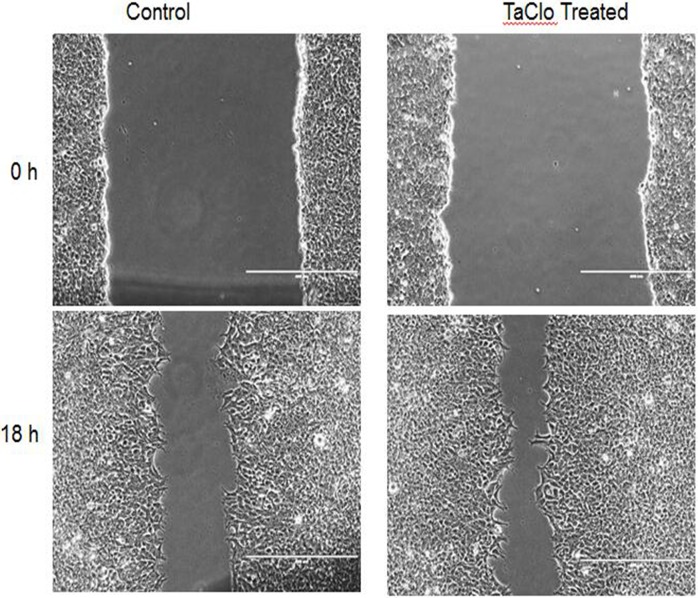
Analysis of cell migration by *in vitro* scratch assay. Images were acquired at 0 and 18 h. Scale bar, 400 μm.

Inhibited expression of tumor suppression genes could disturb the balance between proliferation and apoptosis and enable malignant growth [39]. Therefore, cell proliferation assay was carried out to determine whether TaClo exposure has the ability to regulate cell proliferation. The growth curve was plotted after 6 days of culturing, and the results are shown in Fig 6. Compared with the control, the proliferation rate of TaClo treated cells significantly increased by 33% (P<0.05). Furthermore, the effect of TaClo treatment was also analyzed using a migration assay, and the migration ability of TaClo treated cells was enhanced compared to control cells (Fig 7).

### TaClo exposure enhanced cell anchorage independent growth ability

**Fig 8:**
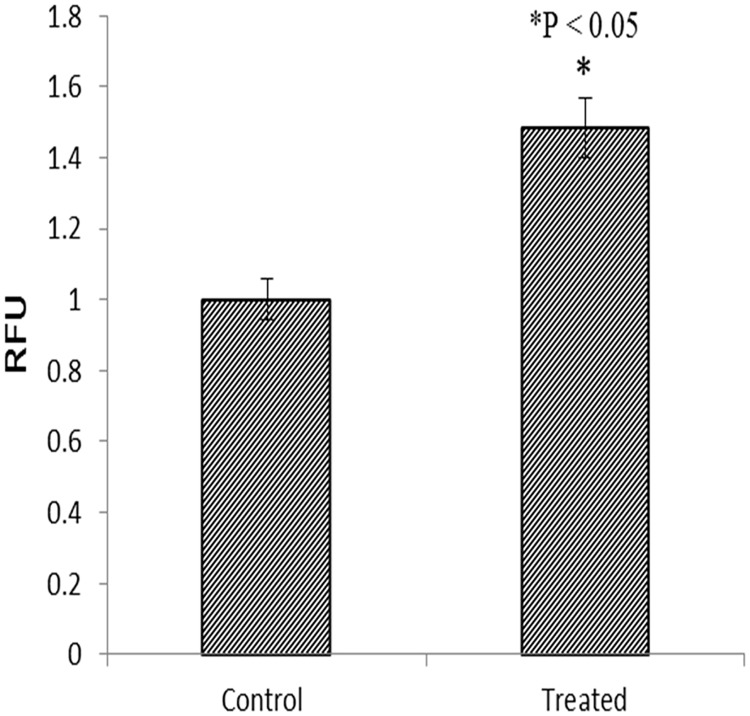
Anchorage independent growth of control and TaClo treated HEK 293 cells. After TaClo treatment, the fluorescence intensities increased around 1.5 fold.

One hallmark of cell carcinogenesis is anchorage independent growth, which is the ability to grow on a solid surface independently [40]. In order to confirm the carcinogenic potential of TaClo, the soft agar colony formation assay was performed in this study by using CytoSelect^™^ 96-well cell transformation assay kit, which is able to detect colonies before they are visible under the microscope and significantly reduce the incubation time. By comparing the fluorescence intensities, TaClo treated cells exhibited significantly increased signals (Fig 8), illustrating the carcinogenic function of low-dose exposure to TaClo.

## Discussion

Current information on the effect of TaClo exposure is limited to the cytotoxic and genotoxic survey as a neurotoxic material, hence there is less awareness of the carcinogenicity of TaClo. Because TaClo is able to penetrate biological membranes passively owing to the high lipophilicity of the CCl_3_ group, there is a concern that TaClo may not only be a neurotoxin, but also have other adverse effect on human health. The DNA methylation modification, including global DNA hypomethylation and promoter CpG island hypermethylation of tumor suppressor gene occurs very early in almost all human cancers and are common epigenetic features of cancer cells [41]. In this study, the changes in DNA methylation and related gene expression were characterized and cell proliferation and migration was evaluated before and after TaClo treatment.

5-methylcytosine (5mC) in the genome can be produced after DNA synthesis in which the carbon-5 position of cytosine obtains a methyl group from S-adenosylmethionine catalyzed by a series of DNA methyltransferases (*DNMTs*) [42]. One of the characteristic patterns of cancer cells is the global DNA hypomethylation, which can change the architectures of chromatin and trigger mutation events. The global DNA hypomethylation observed in this study after exposure to 10 ppb of TaClo for 4 weeks provided essential information on the carcinogenicity of TaClo. The global DNA methylation status is inseparable from both DNA methyltransferases *(DNMT1*, *DNMT3A*, *DNMT3B*) and demethylase (ten-eleven translocation TET proteins). *DNMT1* is involved in maintenance methylation by restoring hemi-methylated sites to full methylation, and *DNMT3A* and *DNMT3B* are responsible for *de novo* methylation [43]. By comparison, *TET* proteins (*TET1*, *TET2*, *TET3*), a family of 2-oxoglutarate and Fe (II)-dependent dioxygenases, play a role in active demethylation by catalyzing 5mC oxidation to a series of derivatives [44, 45]. After TaClo treatment, the transcriptional levels of *TET* genes increased, whereas no significant changes were observed in *DNMTs* genes. Therefore, global DNA hypomethylation might be a result of active demethylation due to increased expression level of *TET* genes.

In general, cancer cells are characterized by global DNA hypomethylation and promoter hypermethylation of tumor suppressor genes. Exposure to carcinogenic substance can induce such a unique methylome [24]. The promoter methylation status of tumor suppressor gene is an important aspect to test our hypothesis that TaClo is carcinogenic to human. As expected, the results from tumor suppressor gene assays showed aberrant promoter hypermethylation of several key tumor suppressor genes in HEK 293 cells after TaClo treatment. Moreover, since *BRCA1* is a well-studied tumor suppressor gene and involved in signal transduction and the repair of DNA damage [46], 11 CpG sites in *BRCA1* promoter were subjected to pyrosequencing. The results of pyrosequencing revealed the formation of some novel methylated CpG sites and enhanced methylation level of methylated CpG sites in TaClo treated samples, providing further evidence that TaClo can induce prompter hypermethylation in tumor suppressor genes.

The most critical effect of hypermethylation in the gene promoter region is associated with transcriptional silencing, and is primarily responsible for the inactivation of tumor-suppressor genes [47]. In agreement with this finding, qRT-PCR results demonstrated that TaClo treatment induced down-regulation of tumor suppressor gene expression by elevating the promoter methylation. The down-regulated tumor suppressor genes were involved in a variety of cellular signaling pathways such as apoptosis, cell adhesion, DNA damage repair, cell cycle and signal transduction. The change in expression of *BRCA1*, *GSTP1*, *PTEN*, and others have been widely documented in various types of human cancers [48].

Previous studies have noted that global DNA hypomethylation can promote oncogenesis, on the contrary aberrant promoter hypermethylation of tumor suppressor genes can repress the expression, and both of these aberrant methylomes can activate proliferation and migration [49]. After 10 ppb TaClo exposure for 4 weeks, few of the critical tumor suppressor genes whose promoters were hypermethylated in the treated cells lost their appropriate adjusting functions in inhibiting cell proliferation and migration. For instance, *GSTP1* expression has been involved in regulating cell proliferation and apoptosis by directly interacting with the c-Jun N-terminal kinase (JNK) [50]. The *PTEN* gene can negatively regulate cell interactions with the extracellular matrix, and can therefore inhibit cell migration [51]. The down-regulated genes, such as *P53* [52], *BRCA1* [53], *GSTP1* [50] can more likely accelerate cell proliferation, and the reduced expression of *CDH1*[54], *BRCA1*[55] and *PTEN*[51] may contribute more to migration. Due to global DNA hypomethylation and the down-regulation of some tumor suppressor genes, the TaClo-treated cells exhibited a higher proliferation rate than the control normal cells. With the ability to evade programmable cell death (apoptosis), uncontrolled cell division and malignant growth are characteristics of cancer cells [56]. The enhanced migration and anchorage independent growth ability of TaClo treated cells observed in this study supports our hypothesis that TaClo is not only neurotoxic but also acts as a carcinogenic substance.

## Conclusion

The carcinogenicity of TaClo was demonstrated in this study by examining the modification of DNA methylation using HEK 293 cells as a model organism. After 4 weeks of exposure to 10 ppb of TaClo, the global methylation decreased by 30%. The transcriptional levels of demethylase (*TET*) were all increased, however we did not observe a significant change in the expression of DNA methyltransferases (*DNMT1*, *DNMT3A*, *DNMT3B*) genes. The unbalanced expression of demethylase and DNA methyltransferases might be a major reason for the global DNA hypomethylation induced by TaClo. Furthermore, the hypermethylation of promoter regions of some tumor suppression genes and the transcriptional repression of these genes were detected in the treated cells. Moreover, compared with the control cells, the TaClo-treated cells manifested higher proliferation rate and enhanced migration as well as anchorage independent growth ability. Taken together, our results show the carcinogenic potential of TaClo by altering the DNA methylation patterns.
